# Nanocellulose Hybrids with Metal Oxides Nanoparticles for Biomedical Applications

**DOI:** 10.3390/molecules25184045

**Published:** 2020-09-04

**Authors:** Madalina Oprea, Denis Mihaela Panaitescu

**Affiliations:** 1 National Institute for Research and Development in Chemistry and Petrochemistry ICECHIM, Splaiul Independentei 202, 060021 Bucharest, Romania; madalinna_09@yahoo.com; 2 Faculty of Applied Chemistry and Materials Science, University Politehnica of Bucharest, Gheorghe Polizu 1-7, 011061 Bucharest, Romania

**Keywords:** cellulose nanofibrils, cellulose nanocrystals, bacterial cellulose, metal oxides, hybrids, bactericide, superparamagnetic, contrast agents

## Abstract

Cellulose is one of the most affordable, sustainable and renewable resources, and has attracted much attention especially in the form of nanocellulose. Bacterial cellulose, cellulose nanocrystals or nanofibers may serve as a polymer support to enhance the effectiveness of metal nanoparticles. The resultant hybrids are valuable materials for biomedical applications due to the novel optical, electronic, magnetic and antibacterial properties. In the present review, the preparation methods, properties and application of nanocellulose hybrids with different metal oxides nanoparticles such as zinc oxide, titanium dioxide, copper oxide, magnesium oxide or magnetite are thoroughly discussed. Nanocellulose-metal oxides antibacterial formulations are preferred to antibiotics due to the lack of microbial resistance, which is the main cause for the antibiotics failure to cure infections. Metal oxide nanoparticles may be separately synthesized and added to nanocellulose (ex situ processes) or they can be synthesized using nanocellulose as a template (in situ processes). In the latter case, the precursor is trapped inside the nanocellulose network and then reduced to the metal oxide. The influence of the synthesis methods and conditions on the thermal and mechanical properties, along with the bactericidal and cytotoxicity responses of nanocellulose-metal oxides hybrids were mainly analyzed in this review. The current status of research in the field and future perspectives were also signaled.

## 1. Introduction

Cellulose is the most affordable, sustainable and renewable resource, which has attracted much attention in the last decades and stimulated researchers to develop cellulose-based materials with novel functions. Cellulosic materials with nanometer size at least in one dimension are referred to as nanocellulose. This nanomaterial is either isolated from plants or synthesized by bacteria. It shows high strength, low density, high crystallinity along with biodegradability and biocompatibility [1,2]. Nanocellulose is a very strong material, with a longitudinal Young’s modulus exceeding 100 GPa and a transverse modulus between 10 and 50 GPa [3,4]. Due to its high stiffness, nanocellulose was largely used as a reinforcing agent for many polymer matrices. Good results were obtained in the case of nanocellulose reinforced biopolymers, due to their inherent low mechanical properties [2,4,5,6]. In addition, nanocellulose may serve as a polymer matrix for organic or inorganic agents in the form of nanoparticles, nanofibers or nanoplatelets [7,8]. Nanocellulose-based materials cover a huge range of applications, from biosensors, energy storage devices and flexible electronics to enzyme immobilization, wound healing, biodegradable packaging, CO_2_ absorbent materials, water purification and oil recovery [2,8]. However, the design of new nanocellulose-based materials for the biomedical field experienced the biggest expansion [9].

Although a valuable material, nanocellulose did not show special electrical, magnetic or antibacterial properties required by some biomedical applications. Metal oxides nanoparticles (MONPs) attracted a high interest due their special optical, electronic, magnetic and antibacterial properties [10]. Zinc oxide (ZnO), copper oxide (CuO), magnesium oxide (MgO) and titanium dioxide (TiO_2_) are intensively studied for healthcare products, biocides, catalysts, electronics, optical devices, biosensors and other cutting-edge applications. The properties of MONPs depend on their size, shape, surface area, crystallinity and stability, which are controlled by the synthesis method and conditions [11]. In general, MONPs may be obtained by physical methods such as ball milling, electrospraying or sputtering and chemical routes such as sol-gel synthesis, hydrothermal method, co-precipitation, chemical vapor deposition or microemulsion technique [11]. The wet chemical methods allow a better control of the size, composition and structure and are generally preferred.

Nanocellulose may acquire new properties by combining with metal oxides. Due to their high surface energy, metal oxide nanoparticles (NPs) have an aggregation tendency when suspended in aqueous media or inserted in polymers [12]. A strategy for improving dispersion involves the use of nanocellulose as a supporting material for the fabrication of metal oxides NPs. Nanocellulose/metal oxides hybrids showing antibacterial, magnetic, sensing properties or improved absorption are required in packaging, wound healing, magnetic resonance imaging (MRI), drug delivery, bio-separation or water cleaning [7,8,9,10]. Metal oxides are preferred in nanocellulose-based antibacterial formulations due to their prolonged release and lack of microbial resistance, which is frequently observed in the case of antibiotics [13]. Indeed, the development of microbial resistance is the main cause for the failure of antibiotics in curing infections [14]. MONPs are efficient against a broad range of bacteria, viruses or fungi due to the release of reactive oxygen species (ROS) which kill microorganisms. More precisely, ROS simultaneously attack the microorganisms on multiple sites leading to their oxidation and death.

A distinct class of metal oxides, superparamagnetic iron oxides nanoparticles with very small size (SPIONs), usually below 15 nm, is intensively studied for magnetically controllable drug delivery systems, cell labeling, biosensors and contrast agents for MRI [14]. SPIONs are non-toxic in small concentration, biodegradable and biocompatible and display a high MRI contrast effect. MRI is a non-invasive high spatial resolution technique for diagnostics, which measures the proton relaxation under an external magnetic field. SPIONs lead to the image contrast by dephasing the proton spin and decreasing of spin–spin relaxation time (T2). For biomedical applications, SPIONs need to be covered by a biocompatible shell to prevent aggregation or degradation and to delay the immune response [15]. Nanocellulose proved to be an excellent biocompatible matrix for SPIONs in MRI applications.

Several reviews on nanocellulose hybrids designed mostly for packaging applications have been already published [7,8,9]. Nonetheless, to the best of our knowledge, no detailed review on nanocellulose/metal oxides intended for biomedical applications was reported so far. In the present paper, the preparation methods, properties and application of nanocellulose hybrids with different metal oxides, ZnO, TiO_2_, CuO, MgO or Fe_3_O_4_, are discussed. Bacterial cellulose (BC) and plant derived cellulose nanocrystals or nanofibers are considered as substrates to enhance the effectiveness of these nanoparticles.

## 2. Nanocellulose—Isolation from Different Sources, Structure and Properties

Cellulose can be isolated from wood, plants, algae, tunicates or agriculture waste biomass by chemical treatments, such as alkali extraction and bleaching, which remove lignin, hemicelluloses and impurities [1,2]. Cellulose contains β-1,4-linked glucopyranose units, each glucopyranose unit bearing three hydroxyl groups. These hydroxyl groups are the source of the high hydrophilicity and biodegradability of cellulose [2]. Their ability to form strong hydrogen bonds provides cellulose with high strength and insolubility in water and usual solvents.

Nanocellulose may be obtained from these cellulose sources by mechanical disintegration (high-pressure homogenization, high power ultrasonication or microfluidization) or by chemical treatments, usually hydrolysis with strong acids (sulfuric, hydrochloric, orthophosphoric or formic) [6,16]. In general, the mechanical disintegration is preceded by chemical or enzymatic pre-treatments with the role of reducing the energy needed for defibrillation. TEMPO-mediated oxidation is considered a clean process, which does not only facilitate the defibrillation process, but also reduces the number of passes and, thus, the energy consumption [17]. Moreover, carboxyl and aldehyde groups are efficiently introduced on the surface of cellulose by TEMPO-oxidation [18,19]. Plasma jet submerged in the liquid suspensions of cellulose was also proposed as an environmentally friendly pretreatment for the defibrillation and functionalization of cellulose [19,20].

**Figure 1:**
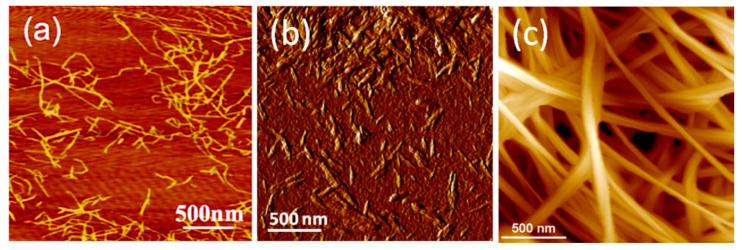
AFM images of (**a**) cellulose nanofibrils (CNF) [23], (**b**) cellulose nanocrystals (CNC) [24] and (**c**) bacterial cellulose (BC) [6].

Nanocelluloses with different characteristics are obtained by these processes: (a) microfibrillated, nanofibrillated cellulose or cellulose nanofibrils (CNF), characterized by a higher aspect ratio and flexibility, are obtained by mechanical treatment as the main step and (b) cellulose nanocrystals (CNC) or (nano)whiskers, with lower aspect ratio and high crystallinity, are obtained by acid hydrolysis. CNFs structure contains more amorphous regions than CNCs due to the milder chemical and mechanical treatments applied for the isolation of nanocellulose that do not alter the fibrous structure [21]. On the contrary, during acid hydrolysis the amorphous regions of cellulose are attacked and disintegrated resulting rod-like rigid high crystalline CNCs [22]. AFM images of CNF and CNC celluloses are shown in Figure 1a,b [23,24].

Several attempts for biomedical applications of CNF and CNC isolated from plants and wood were also reported [25,26,27]. No toxicity in vitro and in vivo was detected for CNF modified by tempo-oxidation and carboxymethylation [26]. Moreover, previous reports have shown that high purity CNF may be applied in wound healing and scaffolding [27]. Similarly, cell culture experiments demonstrated that electrospun cellulose/CNC nanocomposites were nontoxic to human cells, showing rapid cells proliferation on the surface and inside the scaffolds [28].

Nanocellulose may be also synthesized by bacteria and fungi through oxidative fermentation. In particular, nanocellulose is secreted as an exo-polysaccharide from several bacteria such as *Komagataeibacter* (formerly *Gluconacetobacter*), *Agrobacterium*, *Pseudomonas*, *Rhizobium* or *Alcaligenes* [29]. The microbial synthesis route generates a nanofibrillar structure with unique organization, which provides BC high crystallinity, porosity and water uptake capability besides biodegradability and non-toxicity [30]. The most important step in BC formation is the polymerization of glucose, giving β-1→4 glucan chains, followed by the extracellular secretion of these chains from each pore in the cell walls of bacteria. Aggregates of about 1.5 nm in width are formed by the association of adjacent chains. By further combination of the aggregates in protofibrils and subsequently in flat ribbons the BC network is completed (Figure 1c) [6]. Compared to the nanocellulose from plants, algae, marine animals or biomass, biosynthesized cellulose has a higher crystallinity and purity due to the lack of lignin, hemicelluloses, pectin traces and other impurities. To be used in some biomedical applications, the bacterial cellulose membrane should be disrupted by mechanical and/or chemical treatments to provide individual cellulose nanofibers [31] or freeze-dried to obtain 3D nanocellulose networks as foams or aerogels [2,30]. Although cellulose does not readily degrade in the human body due to the lack of cellulolytic enzymes, it has less or even a non-immunogenic reaction. BC has a better biocompatibility than other types of nanocellulose due to its biosynthesis procedure. In addition, it was accepted by FDA as a “generally recognized as safe” (GRAS) material starting from 1992 [32]. In contact with living tissue, it does not cause toxic or allergic side effects and, due to its high porosity, it promotes cellular integration into the cellulose scaffold. All these properties recommend BC for medical applications: soft and bone tissue engineering, wound healing, implants, drug delivery. At the moment, bacterial cellulose is not produced in industrial facilities, however nanocellulose is industrially produced in the form of CNF and CNC.

## 3. Antibacterial Nanocellulose-Metal Oxides Hybrids

The emergence of drug-resistant pathogens is a pressing issue in the biomedical field, especially in wound healing. Multiple studies were conducted towards the development of a novel class of antibacterial materials that meet the mandatory requirements (e.g., biocompatibility, non-toxicity, superior mechanical properties) [33] and also have a bactericidal action that is not based on antibiotics. One approach consists in the use of a natural polymer which ensures the biocompatibility and serves as a solid support for inorganic NPs together with metal or metal oxides nanoparticles (Au, Ag, ZnO, TiO_2_, CuO) as active antimicrobial fillers [34,35,36,37,38,39]. Regarding the natural polymer, bacterial cellulose is a promising option due to its high purity, biodegradability, non-toxicity, high water uptake ability and 3D porous structure that gives it the capacity to retain excess exudates while maintaining an optimal moisture level at the wound site [40].

### 3.1. Bacterial Cellulose/ZnO Hybrids

Zinc oxide nanoparticles (ZnO NPs) are widely used in everyday products such as cosmetics, sunscreens, food packaging and ointments in virtue of their antibacterial properties and ability to effectively absorb UV radiations [41]. The use of ZnO in the biomedical field is encouraged by the fact that the Food and Drug Administration (FDA) currently lists it as a generally recognized as safe (GRAS) material. Photocatalytic cellular membrane disruption and the generation of reactive oxygen species (ROS) are thought to be the main antibacterial mechanisms of ZnO NPs. The antimicrobial efficiency of ZnO NPs is correlated with their size and concentration, higher concentrations and lower particle dimensions generating a stronger bactericidal effect. Zinc is an essential trace element and plays a major role in modulating wound healing phases. Moreover, the slow release of zinc ions at the site of the injury has the potential to enhance wound healing [42]. Therefore, ZnO NPs represent ideal therapeutic agents for inclusion in wound healing mats [40]. There are various ZnO NPs synthesis approaches [43,44]. The chemical route, in either liquid (sol-gel, co-precipitation, water–oil microemulsions, hydrothermal, solvothermal or sonochemical methods) or vapor phase (pyrolysis, inert gas condensation) is most frequently employed because in this case nanoparticles present good surface properties and high purity [45].

#### 3.1.1. Hybrids Obtained by Ex Situ Synthesis of NPs

**Figure 2:**
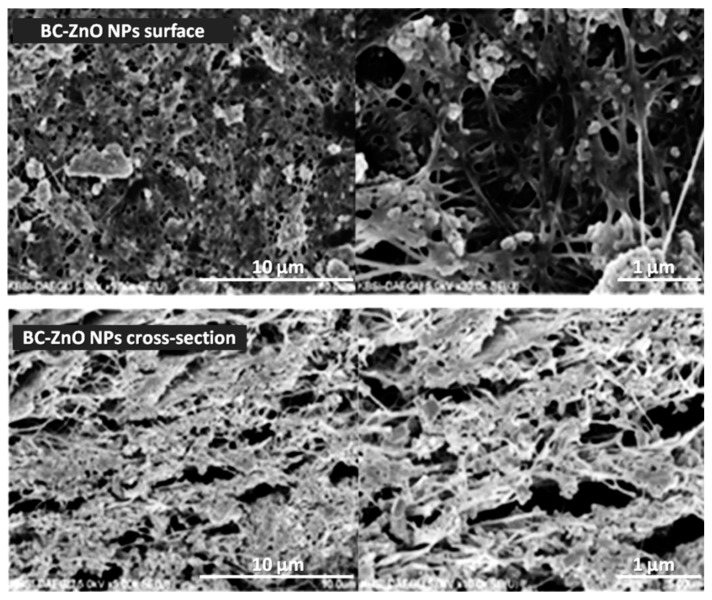
Field emission scanning electron microscopy (FE-SEM) images of surface and cross section of bacterial cellulose/zinc oxide (BC/ZnO) hybrids confirming ZnO nanoparticles (NPs) inclusion in the BC network [47].

ZnO nanoparticles can be separately synthesized and added to BC producing cultures or incorporated in the already formed BC pellicle. However, the cytotoxic effect of the inorganic particles against the BC-producing microorganisms limits the culture addition method and the latter one is often preferred [46]. Khalid et al. [47] prepared bacterial cellulose/zinc oxide (BC/ZnO) hybrids by immersing BC pellicles in a suspension of previously synthesized ZnO nanoparticles and mixing the two components in a shaking incubator at 50 °C for 24 h to promote the dispersion of NPs in the BC network. The nanoparticles were obtained by a simple and efficient method, starting from aqueous solutions of zinc nitrate (Zn(NO_3_)_2_) and sodium hydroxide (NaOH) [48]. The NPs were round to oval in shape and had an average size of approximately 38 nm. Field emission scanning electron microscopy (FE-SEM) images revealed that ZnO NPs were strongly attached to the cellulosic fibers due to the electrostatic interactions between positively charged Zn^2+^ ions and negatively charged hydroxyl groups on the polymeric chains. In addition, they were homogenously distributed, not only on the surface but also inside the BC network (Figure 2), thus indicating that the homogenization in the shaking incubator was effective. During the agar disc diffusion assay, the nanomaterials presented a pronounced growth inhibition effect against some of the most common pathogens involved in burn wounds infections—*Escherichia coli*, *Citrobacter freundii*, *Pseudomonas aeruginosa* and *Staphylococcus aureus*. The wound healing efficiency was investigated using albino BALB/c mice as animal models. An approximate 190 mm^2^ contraction of the wound diameters was observed 15 days post-injury for the BC/ZnO hybrids-treated group and no signs of infection were present. The results were similar to the positive control group treated with silver sulfadiazine, the therapeutic agent currently used for burn wounds [47].

Some issues that may arise when preparing hybrid materials by immersing or blending the polymer with previously synthesized inorganic particles include the particle aggregation tendency and their uneven distribution inside the organic matrix [49,50]. Biocompatible nanomaterials with controlled quantity and distribution of ZnO on dry or humid BC membranes were obtained using matrix assisted pulsed laser evaporation (MAPLE) [51]. The nanoparticles were synthesized by reducing zinc acetate, a zinc oxide precursor, in the presence of ammonia. Transmission electron microscopy (TEM) images revealed that the average nanoparticles size was around 20–30 nm and high resolution TEM (HRTEM) analysis highlighted specific lattice parameters for ZnO with wurtzite crystalline structure. Two types of solvents (water and chloroform) were employed for the MAPLE target preparation in order to study the solvent influence on the particle distribution. The laser deposition process took place in a vacuum chamber, using a pulsed laser system working at a wavelength of 266 nm. A better absorption of the laser source energy by chloroform led to a higher concentration of inorganic particles deposited on BC surface compared to the targets prepared from aqueous dispersions. The mass distribution of inorganic NPs, estimated from atomic force microscopy (AFM) measurements, was approximately 0.28 µg/mm^2^ for the targets prepared in aqueous dispersion and 0.56 µg/mm^2^ for the ones in chloroform. The surface of the hybrids cultured with *Escherichia coli* for 72 h was observed using SEM. Neat BC had no inhibition effect on the bacterial development. Contrarily, only a small number of microorganisms adhered on the surface of the BC/ZnO nanomaterials and their morphology was modified due to the direct action of Zn^2+^ ions on the bacterial cell membrane [51]. Biocompatibility assays on human dermal fibroblasts (HDF) were effectuated only on chloroform-deposited samples considering their enhanced bactericidal effect due to their higher content of ZnO NPs. Just a slight decrease of cellular viability was observed for the BC/ZnO hybrids compared to neat BC during the 72 h test duration. Although previous studies reported that ZnO NPs induce apoptosis in human dermal fibroblasts [52], the good compatibility observed between MAPLE-prepared BC/ZnO hybrids and HDF could be related to the very thin layer (~300 nm) of ZnO NPs on the materials surface, that has the ability to completely inhibit bacterial growth without generating cytotoxicity [51].

An alternative route for the preparation of BC-metal oxides hybrids involves the regeneration of BC from its solutions. Ul-Islam et al. [53] reported the synthesis of regenerated bacterial cellulose (RBC) hybrids with ZnO nanoparticles by dissolving powdered BC in *N*-methylmorpholine-*N*-oxide (NMMO) monohydrate, an organic cyclic polar solvent, considered nontoxic and easily recyclable [54]. ZnO nanoparticles were synthesized using a previously described method [48] and dispersed in the BC solution using ultrasound assisted mixing to prevent aggregation. Next, hybrid films were prepared by casting the solutions using a bar applicator. During solution blending and ultrasonication, the ZnO nanoparticles became attached to the surface and inside RBC matrix as observed in SEM images. Agar disc diffusion and optical density methods were applied to investigate the antibacterial activity of the prepared materials against *Escherichia coli* bacterial strain. As expected, RBC had no antibacterial activity and slightly promoted bacterial growth, whereas RBC/ZnO hybrids had a clear bactericidal effect, the zones of growth inhibition measuring from 34 to 41 mm in diameter. In addition to the bactericidal effect, the nanomaterials were biocompatible showing negligible toxicity towards animal osteoblast cells. These characteristics recommend RBC/ZnO hybrids for biomedical applications such as wound healing and bioelectroanalysis [53].

#### 3.1.2. Hybrids Obtained by In Situ Synthesis of NPs

**Figure 3:**
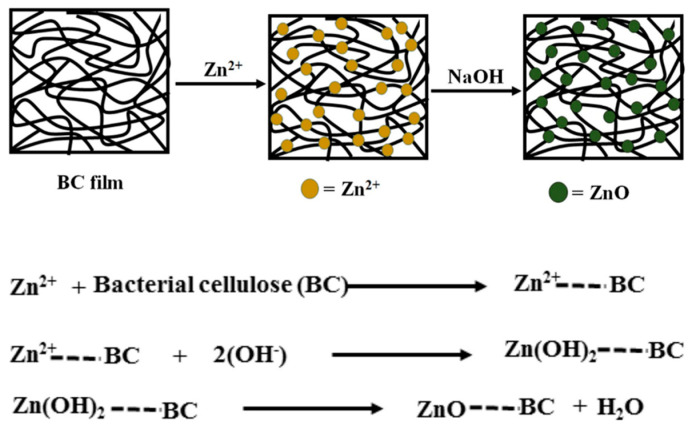
The mechanism proposed for the formation of ZnO NPs inside the BC network [59].

Another technique used to obtain homogenous hybrid materials consists in the in situ synthesis of the inorganic particles using BC as a template [55]. The conversion of Zn^2+^ ions from zinc precursors into ZnO nanoparticles requires a high activation energy [56]. During ex situ synthesis procedures, this energy is provided by the calcination process, which takes place at over 500 °C. However, bacterial cellulose cannot withstand such elevated temperatures and decomposes into a carbon residue [57]. Several strategies for the in situ synthesis of ZnO NPs, directly on the BC pellicle, were developed. For example, Katepetch et al. [55] used ultrasonic-assisted in situ synthesis to produce and simultaneously incorporate zinc oxide nanoparticles into the 3D nanofibrous network of BC. First, bacterial cellulose was impregnated with a zinc acetate solution and Zn^2+^ ions were trapped inside the BC pellicle following the interaction with electron rich oxygen atoms of the polar hydroxyl and ether groups on the surface of cellulose nanofibrils. Afterwards, the pellicles were immersed in ammonium hydroxide (NH_4_OH) solution and ultrasonicated [55]. Cavitation, which consists in the generation and collapse of microbubbles, occurs under the influence of ultrasonic waves and produces elevated pressure and temperature [58] that favors the formation of ZnO NPs. The impregnation time and ultrasonic treatment duration had a significant effect on the size and incorporation percentage of ZnO into BC—a higher impregnation time led to an increased amount of ZnO NPs into the pellicles and a longer ultrasonic treatment resulted in smaller crystal size (54–63 nm) [55]. The nanohybrids exhibited promising antibacterial activity against *Escherichia coli* and *Staphylococcus aureus*, a 99.8% reduction in cell viability being estimated during the colony forming unit assay. These results indicated that the ultrasonication can successfully replace calcination for the simultaneous synthesis and incorporation of antibacterial ZnO NPs in organic matrices. Wahid et al. [59] also obtained BC/ZnO nanohybrid films using a single-pot method. This consisted in the impregnation of BC pellicles in zinc nitrate Zn(NO_3_)_2_ solutions of different concentrations, followed by sodium hydroxide (NaOH) treatment and vacuum drying with a sheet forming instrument at 80 °C for 20 min. Both (ZnNO_3_)_2_ impregnation and alkaline treatment were performed in a shaking incubator for 24 h at room temperature [59]. The hydrophilic nature of BC and its porous structure allowed the migration of Zn^2+^ ions into the 3D cellulosic network until the adsorption equilibrium was reached [60]. Afterwards, Zn^2+^ was converted to ZnO NPs following the interaction with OH^−^ ions from NaOH. A schematic representation of the mechanism proposed for the generation of ZnO NPs inside the BC network is shown in Figure 3 [59]. SEM images showed that the obtained nanoparticles, with size ranging from 70 to 100 nm, had an even distribution inside the polymeric network. During antibacterial assessments on Gram-positive (*Staphylococcus aureus, Bacillus subtilis*) and Gram-negative (*Escherichia coli, Pseudomonas aeruginosa*) bacterial strains, it was observed that higher contents of ZnO NPs in the hybrids were associated with increased diameters of the growth inhibition zones [59]. Moreover, the bactericidal effect was more pronounced against Gram-positive bacteria as a result of their permeable cellular wall that does not restrict the penetration of antimicrobial agents [61,62].

Considering the environmental concerns nowadays, green technologies that reduce the amount of chemicals used for the synthesis of ZnO NPs were explored. Solution plasma processing (SPP) was found to be an effective ecofriendly method for the preparation of ZnO/BC composites without the addition of a reducing reagent (e.g., NH_3_, NH_4_OH, NaOH) [63]. Solution plasma (SP) is an electrical discharge phenomenon that takes place at room temperature, in a liquid medium (e.g., aqueous solutions or organic compounds), the result being an atmospheric non-equilibrium plasma [64]. The chemical species generated during SPP—anions (O^−^, OH^−^), radicals (H, O, HO) and free electrons, could have the ability to initiate the conversion of metal ions to metal oxide nanoparticles [65]. This procedure was successfully applied for the synthesis and deposition of ZnO NPs into BC pellicles. Bacterial cellulose was saturated in methanol for 72 h and subsequently immersed for 24 h in zinc nitrate or zinc acetate solutions of various concentrations [63]. The saturated pellicles were placed in a SP glass reactor containing methanol, and the plasma treatment was performed for 1 h. For comparison purposes, some pellicles were prepared with NH_4_OH as a reducing reagent. According to SEM-EDX analysis, SPP and the classical chemical reduction method produced similar quantities of ZnO NPs but the ones obtained by SPP were more homogenously dispersed inside the polymeric network. The type of ZnO precursor influenced the inorganic nanoparticles morphology and their interactions with BC nanofibers [63]. Moreover, FT-IR analysis revealed that the increase of Zn^2+^ precursor concentration generated superior Zn^2+^-BC and ZnO-BC interactions. The results from both disc diffusion and colony counting method showed strong antibacterial activity against *Staphylococcus aureus* and *Escherichia coli*, thus recommending the nanocomposites for applications in wound healing and water disinfection applications [63].

**Figure 4:**
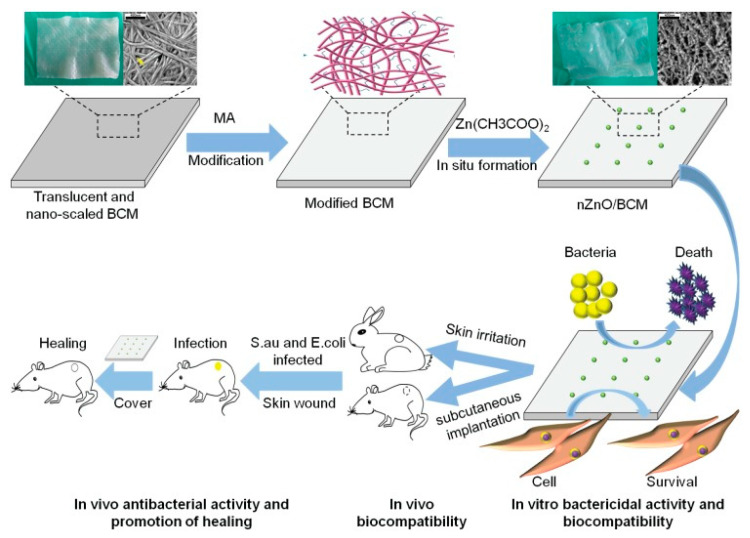
Schematic representation of the synthesis procedure and biological activity of the BCM/ZnO hybrids [40].

The in situ synthesis of ZnO NPs is highly dependent on the ability of the supporting material to adsorb Zn^2+^ ions from the zinc oxide precursor solution. Metal ions are adsorbed at the active sites level, more specifically, the surface functional groups [66]. It was reported that carboxylic groups can act as proton donors and ion exchange sites [67]. Previous study showed that the introduction of carboxyl groups to cellulose generates a significant increase in the adsorption capacity of copper, cadmium and lead ions [68]. Moreover, the electrostatic repulsions that occur among negatively charged carboxylate ions allow a homogenous dispersion of individual cellulose fibers in water [69]. A method for fabricating carboxyl modified bacterial cellulose consists in 2,2,6,6-tetramethylpiperidine-1-oxyl radical (TEMPO) mediated oxidation that leads to the replacement of primary hydroxyl groups of cellulose with carboxyl groups [70]. Likewise, carboxyl groups may be introduced to cellulose via anhydrides. In particular, BC membranes (BCM) were modified with maleic anhydride resulting carboxylated and crosslinked BC membranes (mBCM) that were used as template for the in situ synthesis of ZnO NPs [40]. Nanoparticles were generated by the reduction of zinc acetate with different concentrations of sodium hydroxide in anhydrous ethanol medium. The resulting ZnO/BCM bionanomaterials were freeze-dried and dried again for 1 h at 120 °C before characterization (Figure 4) [40]. It was found that more than 50% of the ZnO NPs were released from the unmodified BCM during immersion in PBS, while less than 10% were released from the mBCM modified membranes. ZnO/mBCM hybrids presented a high porosity and uniformly distributed NPs in the BCM matrix. This structural characteristic was associated with an enhanced water vapor transmission rate (WVTR) for ZnO/mBCM compared to neat BCM [40]. WVTR is an important parameter for wound dressings and values between 2500–3000 g/m^2^/day are required to prevent wound scabbing due to dehydration or bacterial infections caused by excess moisture [71]. Excellent antibacterial activity is also a mandatory feature of an ideal wound dressing [33]. Even if higher doses of ZnO NPs are associated with an enhanced bactericidal action, the amount of antimicrobial particles loaded in the polymer should be carefully adjusted to prevent the occurrence of cytotoxic effects [72]. According to the results obtained from the cytotoxicity tests on mouse fibroblasts, skin irritation tests on New Zealand white rabbits and antibacterial assessments on *Staphylococcus aureus* and *Escherichia coli*, a 5 wt% ZnO NPs content in mBCM was considered optimal. This nontoxic ZnO/mBCM bionanomaterial that showed antibacterial activity and good biocompatibility, leading to rapid re-epithelialization and wound closure (Figure 3), was proposed as an efficient antibacterial wound dressing [40].

Taking into account the results obtained during the presented research studies, it might be concluded that the antibacterial activity of BC/ZnO hybrids generally depends on the concentration and type of Zn^2+^ precursor, the method used to synthesize ZnO NPs—which influences their size and morphology, the technique used to prepare the nanomaterials and the type of bacteria, Gram-positive ones being more sensitive to the antibacterial action of ZnO NPs.

### 3.2. Bacterial Cellulose/TiO_2_ Hybrids

BC-based antibacterial nanomaterials were also obtained by incorporating titanium dioxide nanoparticles (TiO_2_ NPs) into the cellulosic network. Titanium dioxide nanoparticles present great antibacterial and photocatalytic potential especially when they are comprised from mixed crystalline phases of anatase and rutile [73,74]. Due to their chemical stability, non-toxicity and UV blocking ability, they were included in many consumer products, such as food additives (E171), toothpaste, medicines, cosmetics and sunscreens [74]. Studies showed that these properties are well maintained even after incorporation in natural polymeric matrices [75]. BC-TiO_2_ interactions consist of physical adsorption on the surface of nanofibers or the formation of hydrogen or covalent bonds at the site of free hydroxyl groups in the cellulose macromolecules [76]. Multifunctional materials, based on BC and TiO_2_ nanoparticles were prepared by Brandes et al. using an ex situ sol-gel method [77]. Summarily, BC hydrogels were immersed in an aqueous dispersion of TiO_2_ and agitated in an orbital shaker for 3 h at 30 °C, thus allowing the nanoparticles to be retained in the gaps between the cellulosic nanofibers. The first clue of the successful incorporation of NPs in the BC hydrogels was their color transition from translucent to opaque after impregnation with TiO_2_. The hybrid hydrogels were freeze-dried and characterized by SEM, EDX and FT-IR analysis. SEM images showed a high density of TiO_2_ NPs on the surface of the BC nanofibers. The occurrence of molecular interactions and adhesion between the inorganic NPs and the cellulosic support was confirmed by O-H, C-OH and C-O-C peaks shifting in the FT-IR spectrum [77].

Khan et al. estimated that the antimicrobial properties of TiO_2_ may reduce the risk of bacterial contamination of newly formed tissues when used in tissue engineering applications [78]. TiO_2_ NPs were synthesized by drop wise addition of TiCl_4_ in benzyl alcohol under nitrogen flow. High resolution transmission electron microscopy-selected area electron diffraction (HRTEM-SAED) images confirmed the formation of highly crystalline NPs with very small dimensions (20–30 nm) and lattice planes corresponding to the anatase phase of TiO_2_. Powdered BC was dissolved in NMMO/water solvent system (RBC) and the inorganic NPs were incorporated using ultrasound assisted mixing. The RBC/TiO_2_ films were prepared by solution casting. EDX analysis demonstrated the presence and homogenous dispersion of NPs inside the polymeric matrix. An extensive biological characterization was performed to determinate the suitability of the obtained nanomaterials for the intended applications. It was observed that the microbial development was strongly inhibited by both TiO_2_ NPs and RBC/TiO_2_ hybrids, the bactericidal effect of plain TiO_2_ being more pronounced. The antibacterial mechanisms were determined using established fluorometric assessments (ROS mediated oxidation of dichloro-dihydro-fluorescein diacetate and in vitro glutathione oxidation). They were related to the decomposition of bacterial cellular membranes by the highly reactive ROS generated by the NPs and oxidation of the thiol groups from amino acids present in bacterial cells. The nanomaterials also showed a good biocompatibility, their entire surface being uniformly covered by animal fibroblast cells after 7 days of incubation. More than that, the 3-(4,5-dimethylthiazol-2-yl)-2,5-diphenyltetrazolium bromide (MTT) test showed only a negligible decrease in cellular proliferation for RBC/TiO_2_ compared to neat RBC. The results obtained during this study provide a strong foundation for the future usage of RBC/TiO_2_ nanomaterials in biomedical applications particularly as antibacterial scaffolds or wound healing mats [78].

### 3.3. Bacterial Cellulose/CuO Hybrids

Similar to TiO_2_ NPs, nano-sized CuO presents good photocatalytic and photovoltaic properties due to the narrow band gap in its crystal structure. More than that, Cu and CuO nanoparticles were found to have an excellent growth inhibition effect even at low concentrations against fungi, algae, yeasts, Gram-positive and Gram-negative bacterial species [60,79]. There are a variety of techniques that can be applied to synthesize CuO nanostructures (CuO NSs), the most known being the chemical methods such as sol-gel, microemulsions, sonochemical, hydrothermal and alkoxide-based preparation [80].

**Figure 5:**
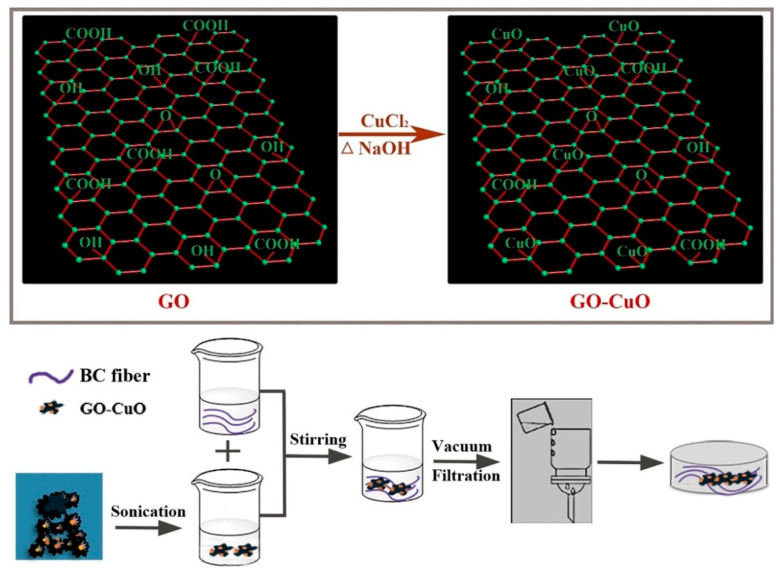
Workflow followed for the development of BC/GO-CuO films [13].

In a recent study by Xie et al. [13], BC/CuO hybrid films were prepared by incorporating the inorganic component into the BC matrix through homogenization blending. CuO nanosheets with variable lengths (50–200 nm) and widths (20–50 nm) were uniformly grown on graphene oxide (GO) platelets. Due to the various functional groups on its surface (e.g., hydroxyl, carboxyl, epoxide), GO provides nucleation sites for an efficient in situ growth of CuO nanostructures [81]. Moreover, GO can keep CuO NSs well dispersed in aqueous suspensions [82]. For the preparation of GO-CuO complexes, cupric chloride (CuCl_2_) was incorporated into an aqueous graphene oxide suspension by magnetic stirring at 100 °C for 60 min [13]. Afterwards, NaOH was slowly added for the conversion of the precursor into CuO nanosheets. The formed precipitate was separated by centrifugation, dried and ground to get GO-CuO powder. BC/GO-CuO nanomaterials were prepared by mixing aqueous suspensions of GO-CuO with BC slurries followed by drying using a sheet forming instrument (Figure 5). During the antibacterial assays performed on *Staphylococcus aureus* and *Escherichia coli*, it was observed that the BC/GO-CuO nanomaterials were more efficient against Gram-positive bacterial strains than that without GO. In addition, BC/GO-CuO hybrid films had better results in terms of microbial growth inhibition compared to BC/CuO films, thus indicating a synergistic bactericidal effect between GO and CuO. A possible mechanism of the antimicrobial action could be related to the direct contact of bacterial cells with the sharp GO-CuO nanostructures. The disruption of cellular membrane integrity caused by this interaction generates a surface collapse accompanied by cellular deformation and the increased production of ROS leads to bacterial cell death. The nanomaterials were non-cytotoxic against mouse embryonic fibroblasts cells (NIH-3T3), a slight increase of cellular viability (compared to the control group) being indicated by the MTT assay results for the group treated with BC films containing 10% GO-CuO. In virtue of their good biocompatibility and antimicrobial activity, the BC/CuO-GO films could represent a new generation of hybrid materials for applications in the biomedical field [13].

### 3.4. Bacterial Cellulose/MgO Nanohybrids

MgO nanoparticles are very interesting for biomedical applications because they are non-toxic in low concentrations (under 250 μg·mL^−1^) [83], very stable, show antibacterial activity, high thermal conductivity and have very good dielectric properties [84,85]. MgO is classified as a generally recognized as safe (GRAS) ingredient by the US FDA [86]. MgO NPs can be synthesized by laser ablation, microemulsion method, hydrothermal synthesis, sol-gel, wet chemical reactions, microwave or ultrasound assisted synthesis [84]. In addition to conventional methods, biochemical methods are increasingly developed for the synthesis of inorganic nanoparticles [86,87]. In particular, natural extract of *Dalbergia sissoo* and water were used as reducing agent and solvent instead of harmful compounds for the synthesis of MgO NPs [87]. One of the most important uses of MgO NPs is as an antibacterial nanomaterial for biomedical application. In particular, MgO-BC nanohybrids were obtained by in situ co-precipitation methods and ex situ incorporation of MgO-NPs in the BC membranes [84]. BC membranes were suspended in a magnesium acetate tetrahydrate (Mg(CH_3_COO)_2_·4H_2_O) precursor solution. Ammonia was added as a precipitating agent and polyethylene glycol as a surfactant. The mixture was kept under continuous agitation for 3 h at 70 °C. The nanohybrids were dried at 80 °C for 18 h and then at 180 °C for 3 h resulting MgO-BC hybrids. In the ex situ method, previously obtained MgO NPs were incorporated in BC membranes by immersion in water under ultrasonic irradiation followed by drying and calcination in the same conditions as in the in situ process [84]. The release tests using an agar disk diffusion method showed a good antibacterial activity for all types of nanohybrids, however, the ex situ synthesized nanohybrid had the highest antimicrobial activity against both *Staphylococcus aureus* and *Escherichia coli*.

### 3.5. Cellulose Nanocrystals/Metal Oxides Hybrids

**Figure 6:**
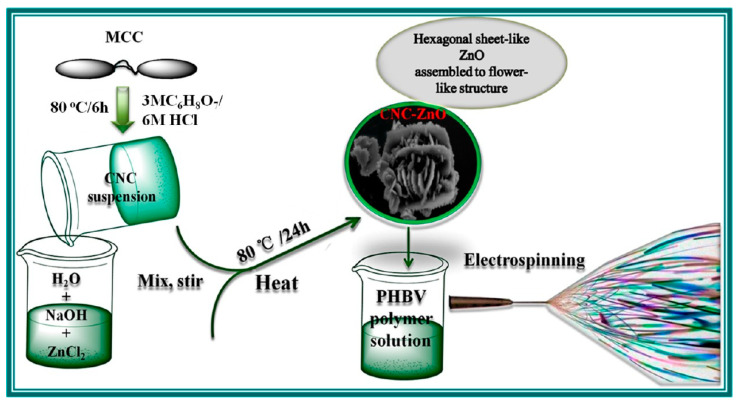
Schematic representation of the CNC-ZnO nanohybrids preparation procedure and the poly(3-hydroxybutyrate-co-3-hydroxyvalerate) (PHBV)/CNC-ZnO electrospinning process [88].

Owing to the electrostatic interactions between cellulose nanocrystals and zinc oxide, sheet-like CNC-ZnO nanohybrids were successfully developed by a one-step hydrothermal method [88]. CNCs were prepared by mixing microcrystalline cellulose (MCC) with an acid solution consisting of 90% citric acid and 10% HCl, at 80 °C for 6 h. The final CNC suspension (neutralized with NH_4_OH and washed with distilled water) was added in an aqueous solution of ZnCl_2_ containing NaOH as precipitating agent and homogenized at room temperature. The obtained CNC-ZnO nanohybrids were introduced into poly(3-hydroxybutyrate-co-3-hydroxyvalerate) (PHBV) using the electrospinning process (Figure 6).

The presence of zinc, carbon and oxygen in the EDX spectrum of hybrid fibers indicated the successful loading of CNC with ZnO NPs. In addition, FE-SEM images revealed that the morphology of CNC-ZnO nanohybrids varied from nanosheets to flower-like structures depending on the concentration of Zn^2+^ ions. The insertion of CNC-ZnO into the PHBV matrix provided it a UV-blocking ability for both UVA (99.72%) and UVB (99.95%) and excellent antimicrobial activity against *Escherichia coli* and *Staphylococcus aureus* [88].

### 3.6. Cellulose Nanofibers/Metal Oxides Hybrids

Cellulose nanofibers were also modified with metal oxide nanoparticles to obtain hybrid materials with improved characteristics. For example, copper and copper oxide coated cellulose nanofibers (CNFs) presented promising antimicrobial properties and biocompatibility [89]. Cellulose nanofibrils were obtained from *Colocasia esculenta* stems by successive bleaching, alkaline treatment, glacial acetic acid hydrolysis and abundant washing. Afterwards, the CNFs were dispersed in ethanol and mixed with copper acetate at room temperature for 2 h. A green reductive technique using an alcoholic extract of *Terminalia chebula* fruit instead of toxic NaBH_4_ was proposed for the reduction of copper salts, assuming that the polyphenol compounds from the fruit extract may form complexes with Cu^2+^ ions, thus reducing Cu(CH_3_COO)_2_. The copper-coated CNFs were freeze-dried before characterization. UV–Vis absorption bands indicated a mixture of Cu and CuO in the hybrids, aspect also highlighted by FT-IR and XRD. The hybrids exhibited strong antimicrobial effects when tested against *Escherichia coli*, *Staphylococcus aureus* and *Candida albicans*. At low concentrations of Cu and CuO (3 or 5%), the nanohybrids induced no degradation of the structural integrity of bovine serum and showed good biocompatibility with peripheral blood mononuclear cells, however, cell death was observed at a higher amount of metal oxide [89,90].

## 4. Nanocellulose Hybrids with Magnetic Nanoparticles

Hybrids consisting of cellulose and magnetic particles have attracted great interest in the biomedical field due to their advantages such as biocompatibility and biodegradability [91]. Iron oxides and, in particular, magnetite (Fe_3_O_4_) show superparamagnetic properties, high stability, low cost, good biocompatibility and low toxicity which recommend them for magnetic biocomposites. Magnetic Fe_3_O_4_ nanoparticles (Fe_3_O_4_ NPs) have a demonstrated efficiency in magnetic resonance imaging, drug delivery, bio-separation, catalysis and wastewater cleaning. Their efficiency as well as physical and chemical properties are influenced by their morphology, size and structure [92]. Currently applied techniques to synthesize MONP include thermal decomposition, co-precipitation and hydrothermal methods. Co-precipitation is usually preferred due to its simplicity, low temperature, time-saving, low cost and high quality of resulted iron oxide NPs [93].

In addition, paramagnetic behavior, nanocellulose hybrids with magnetic nanoparticles are attractive due to their improved optical, antibacterial, conductive and mechanical properties. However, some of these properties are obtained for high concentration of MONPs, which raises the problem of nanoparticle aggregation [94]. The agglomeration of MONPs may compromise the magnetic, optical and mechanical functions of nanocomposites, therefore, the preparation and processing of these nanomaterials are challenging tasks.

### 4.1. Bacterial Cellulose/Iron Oxides Hybrids

**Figure 7:**
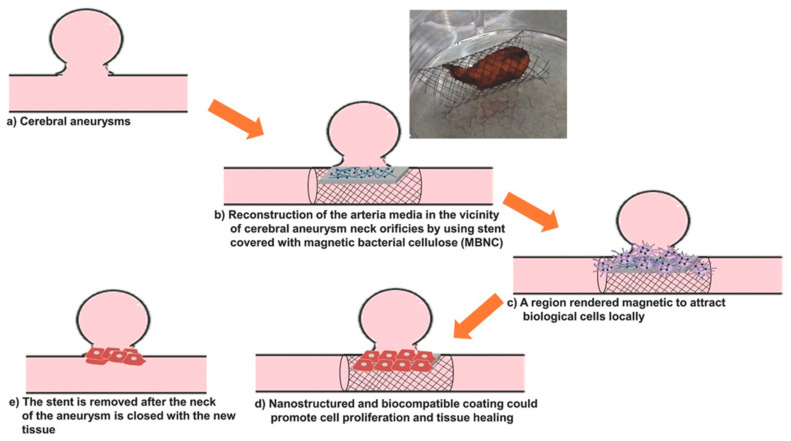
Stages of the blood vessel wall reconstruction process using stents covered with BC/Fe_3_O_4_ hydrogel membranes; photograph of a Nitinol stent covered with magnetic BC, showing good adhesion properties [95].

Cerebral aneurysms are the most critical events in cerebral trauma and surgical conventional treatments have low success rate [95]. Pavon et al. developed an alternative technique for neuro-endovascular reconstruction [95,96,97]. They used a co-precipitation-based method to functionalize bacterial cellulose hydrogel with Fe_3_O_4_ NPs. The materials were designed as coatings for the surface of metallic stents used for the reconstruction of tunica media tissue after a cerebral aneurysm. This novel process consists of arterial media reconstruction by using a stent covered with magnetic BC. Magnetic stimulation is used to orient magnetized endothelial cells, derived from the arterial wall or provided externally via a catheter, to the regions along the outer side of the BC/Fe_3_O_4_-covered stent. This method allows the growth of a new tissue over the device which closes the aneurysmal neck defect (Figure 7) [95]. Cellulose functionalization was performed by impregnating BC membranes with FeCl_3_·6H_2_O and FeCl_2_·4H_2_O solutions at 80 °C, under nitrogen flow and vigorous stirring accompanied by ammonium hydroxide (NH_4_OH) addition to form Fe_3_O_4_ NPs inside the BC [96]. The surface of magnetic NPs used to develop magnetic materials for tissue regeneration should be modified for increased biocompatibility because plain magnetic NPs could cause genotoxicity and cells necrosis [97]. Different solutions were tested for improving biocompatibility. In one attempt, oleic acid was added into the ferrofluid used for BC impregnation during the heating stage [97]. SEM images indicated that the magnetic NPs had an aggregation tendency, most likely due to the polar nature of fatty acid functional groups on their surface. In other attempts, polyethylene glycol (PEG), polyethyleneimine or citric acid were used to coat magnetic materials [95]. The nano-mechanical properties of neat BC and BC/Fe_3_O_4_ were evaluated using in situ nano-indentation measurements in hydrated state. The stiffness range of the BC/Fe_3_O_4_ hybrids (0.0025–0.04 GPa) was close to the values measured for both large arteries and veins of human cerebral vessels. A residual elastic straining effect similar to the one characteristic to biological tissues (e.g., tendons, blood vessels, ligaments) was also observed [97].

Flexible magnetic BC nanohybrids were also obtained by in situ synthesis of Fe_3_O_4_ NPs using ultrasonic irradiation and PEG as a coating polymer [98]. The results showed that ultrasonication and PEG ensured the homogeneous dispersion of Fe_3_O_4_ NPs in the BC network. Moreover, the magnetic BC membranes obtained by this method showed a saturation magnetization of 40.58 emu/g and good mechanical properties.

### 4.2. Cellulose Nanocrystals/Iron Oxides Hybrids

Similarly to other metal oxides nanostructures [12], Fe_3_O_4_ NPs can easily aggregate and oxidize in aqueous or oxygen environments, which limit their applications. To overcome these disadvantages, the surface of magnetic NPs can be modified with functional materials (e.g., mesoporous silica) to obtain core–shell structures with improved stability [99]. Another technique consists of the synthesis of Fe_3_O_4_-grafted cellulose nanocrystals. The negative charges, introduced in cellulose nanocrystals structure during acid hydrolysis preparation process, generate an electrostatic repulsion among CNC-Fe_3_O_4_ particles, hence giving them the ability to effectively disperse in aqueous media [100].

**Figure 8:**
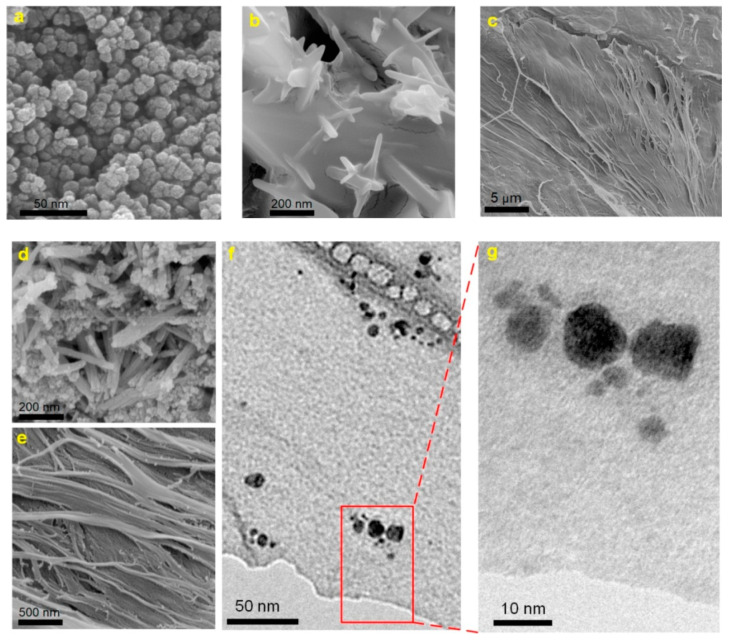
FE-SEM images of (**a**) Fe_3_O_4_ NPs, (**b**,**c**) CNC-PCA and (**d**,**e**) CNC-PCA/Fe_3_O_4_ hybrids, (**f**,**g**) TEM images of CNC-PCA/Fe_3_O_4_ hybrids [104].

MRI is a non-invasive clinical diagnostic technique used for anatomical imaging of soft body tissues. Contrast agents (CAs) are used to improve the image quality by shortening the relaxation time of water protons, thus increasing the MRI sensitivity [101]. Positive (T1) contrast agents reduce longitudinal relaxation time and produce brighter images, while negative (T2) ones shorten transverse relaxation time, resulting in darker images. The sensitivity of a contrast agent is defined by its relaxivity parameters (longitudinal—r1 and transverse—r2). The development of high-relaxivity CAs is desirable because they provide contrast enhancement at lower doses compared to low-relaxivity compounds, therefore the potential toxic effects are reduced [101,102,103]. Nanocellulose in the form of cellulose nanocrystals was used in combination with ultra-small superparamagnetic iron oxide nanoparticles (USPIONs) to develop a novel T1-T2 contrast agent [104]. Cellulose nanocrystals (CNC) were isolated from cotton linters using the acid-hydrolysis method, and incorporated in poly(citric acid) (PCA) to produce a biocompatible, dispersible and stable substrate. USPIONs magnetic nanoparticles were synthesized by thermal decomposition of iron (III) acetylacetonate precursor and loaded on the hydrophilic CNC-PCA substrate. To obtain the CNC-PCA/Fe_3_O_4_ nanohybrids, predetermined quantities of USPIONs and CNC-PCA were separately dispersed in distilled water and ultrasonicated, then mixed for 24 h and dried in a vacuum oven [104]. FE-SEM images showed that the spherical Fe_3_O_4_ NPs were well dispersed on the CNC-PCA surface and no aggregations were observed (Figure 8). The particle size distribution analyzed by dynamic light scattering showed that the average hydrodynamic size of Fe_3_O_4_ NPs and CNC-PCA/Fe_3_O_4_ was 13.2 and 12.0 nm, with a polydispersity index of 0.12 and 0.34.

**Figure 9:**
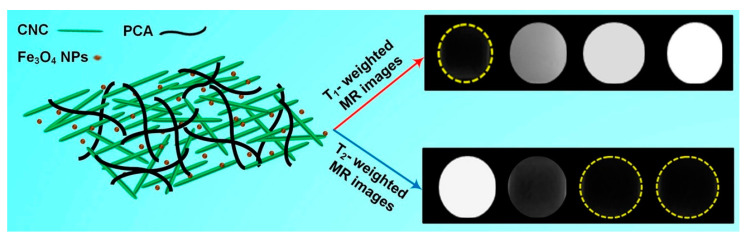
Schematic representation of the CNC-PCA/Fe_3_O_4_ structure; T1- and T2-weighted MRI images of CNC-PCA/Fe_3_O_4_ at increasing (left to right) Fe concentrations (0, 0.1, 0.3, 0.5 mM) [104].

The XRD pattern of CNC-PCA/Fe_3_O_4_ showed all the diffraction peaks corresponding to the crystal planes of Fe_3_O_4_ and two new broad peaks at 2θ = 14.9° and 22.1° associated to (110) and (002) planes in the structure of cellulose. The high saturation magnetization value (52.2 emu·g^−1^) and good relaxivity parameters r1 (13.8 mM^−1^·s^−1^), r2 (96.2 mM^−1^·s^−1^), obtained at 3.0 T magnetic field strength, demonstrated that the hybrids could be used successfully as dual MRI contrast agents (Figure 9). In addition, a higher iron concentration was associated with an enhanced signal intensity of T_1_-weighted images (brighter images) and a reduced signal intensity on T_2_-weighted images (darker images), the contrast being improved in both cases. In vitro cellular uptake was performed using inductively coupled plasma optical emission spectroscopy and in vitro cytotoxicity to HeLa cell lines was also investigated. The results revealed an appropriate cellular uptake, excellent biocompatibility and low toxicity, characteristics which make the CNC-PCA/Fe_3_O_4_ nanohybrids promising for the intended biomedical application [104].

Versatile magnetic materials based on cellulose nanocrystals and cobalt ferrite (CoFe_2_O_4_) were obtained by in situ synthesis of the inorganic nanoparticles on CNC support [105]. CNCs with length of approximately 150 nm were obtained from dry cotton by acid-hydrolysis. The magnetic nanomaterials were synthesized by treating CNC aqueous dispersions with precursor salts-ferrous (II) sulfate heptahydrate (FeSO_4_·7H_2_O) and cobalt chloride (CoCl_2_), followed by heat treatment. After the addition of the precipitating agents, NaOH and KNO_3_, the dispersion changed its color to brown, this being an indicator of CoFe_2_O_4_ particles growth. CNC-CoFe_2_O_4_ nanohybrids were further tested either as magneto-responsive dispersions or as precursors for self-standing films or composites nanofibers. The films could find applications in packaging and magnetic shielding and the composite nanofiber mats could be considered for magnetic separation procedures [105]. The amount of inorganic nanoparticles in CNC-CoFe_2_O_4_ nanohybrids was verified by the residue at 800 °C in TGA measurements. FE-SEM-EDX analysis of CNC-CoFe_2_O_4_ dispersion showed that the inorganic NPs were mainly spherical with diameter of approximately 10–40 nm. The hybrid dispersion was stable, CNCs being able to function as a nucleation site for the inorganic particle growth and also as a stabilizing network. Preliminary studies regarding the utilization of the aqueous CNC-CoFe_2_O_4_ dispersion in magnetic hyperthermia were conducted and an increase with 8 °C of the temperature (from 24 to 32 °C) was achieved in 40 min. This effect may be improved by using a higher magnetic NPs loading [105].

## 5. Influence of Metal Oxide NPs on the Properties of Cellulose Nanohybrids

A summary of the nanocellulose-metal oxide nanocomposites studied for biomedical applications is presented in Table 1. The antibacterial or magnetic properties of nanocellulose-metal oxide nanohybrids were presented in the previous chapters along with the methods of obtaining these nanohybrids. However, MONPs have also an important influence on their thermal and mechanical properties. The thermal behavior of nanocellulose composite with MONPs is important for the biomedical applications of these nanohybrids. BC shows a major degradation step between 250 and 375 °C, with a maximum degradation rate temperature around 320 °C [30,53,59]. This is due to the dehydration, depolymerization and decomposition of glucose units. 

The MONPs are highly thermostable inorganic nanomaterials and their incorporation in nanocellulose might increase the thermal stability. However, different effects of MONPs on the thermal stability of nanocellulose depending on their concentration in the nanohybrids and the composites preparation method were reported. Although characterized by a high thermal stability, metal oxides are sensitive to oxygen in the air, so that thermogravimetric analysis was in general carried out in nitrogen flow.

A decrease of the thermal stability of BC with more than 25 °C was noticed for BC/ZnO nanocomposite films prepared by in situ wet chemical synthesis of NPs [59], however, this decrease was not influenced by the concentration of ZnO NPs in the nanocomposites (between 5 and 34%). Wahid et al. considered that the faster degradation of the BC/ZnO nanohybrids compared to original BC was due to the catalytic activity of ZnO NPs which assisted the cross-linking breakdown in the cellulose network [59]. A similar decrease was observed for BC/ZnO nanohybrids prepared by SPP assisted synthesis and deposition of ZnO NPs into BC pellicles [63]. The influence of the in situ vs. ex situ synthesis of MgO NPs on the thermal stability of BC nanocomposites was highlighted by Mirtalebi et al. [84]. They showed that a higher decrease of the thermal stability was noticed when MgO NPs were obtained by in situ methods. This was explained by the stronger interactions of NPs with the BC membrane which led to disruption of BC crystalline structure [84].

A slight increase of thermal stability with 5 and 10 °C was reported in the case of BC/ZnO (1 and 2% NPs) nanocomposites compared to neat BC [47]. The nanocomposites were obtained by ultrasound assisted mixing of BC-NMMO solution with ZnO NPs. The authors consider that ZnO NPs behave as a barrier in the nanohybrids by absorbing the heat and slowing down the degradation of BC [53]. A significant increase of thermal stability, with more than 50 °C, was noticed in the case of BC/Fe_3_O_4_ nanocomposites with a high proportion of magnetic nanoparticles [98]. The composition and preparation conditions influenced the thermal behavior. Thus, the method involving ultrasonication led to a smaller increase of the thermal stability than the one without ultrasound irradiation due to the disruption of the BC network that decreased its crystallinity [98].

The mechanical properties of the nanocellulose-MONPs hybrids intended for wound dressings or other biomedical applications are very important because they must show both flexibility and mechanical strength to properly protect the wound from damage or collision [40]. In general, the incorporation of MONPs in nanocellulose networks leads to the increase of the mechanical properties of hybrids. An increase of the tensile strength at break with about 20% for BC-ZnO with 1 and 2% ZnO and of the Young’s modulus with 37.5 and 62.5% for the same hybrids, compared to plain BC, was reported by Ul-Islam et al. [53]. This noticeable increase was explained by the covalent/hydrogen bonding occurring between ZnO and OH groups of cellulose, which increases the toughness and limits the chains mobility, thus increasing the strength of the hybrids [53]. This rigidity and diminished mobility of the polymer chains have as result a slight decrease of the tensile strain and a lower flexibility. Similarly, a two-fold increase of the Young’s modulus and an increase with about 30% of the tensile strength without a decrease of flexibility were reported for a carboxylated BC-ZnO hybrid [40]. These properties indicate a flexible and strong material, suitable for wound dressings.

Recent work on BC-MgO nanohybrids highlights the influence of the method used to synthesize the NPs and that to obtain the hybrid (in situ or ex situ, chemical or sonochemical) on the mechanical properties [84]. The effect of MgO NPs on the mechanical properties of the ex situ synthesized nanohybrids was not significant; however, a maximum increase of the Young’s modulus with 72% and of the tensile strength with 30% was noticed for BC-MgO nanohybrids obtained by the in situ methods compared to BC. The uniform distribution of MgO NPs in the whole BC network and increased interactions were considered to cause the improvement of the mechanical properties. On the contrary, the dispersion of MgO NPs only on the surface of cellulose membrane and agglomerations do not modify or even decrease the mechanical properties of the nanohybrids [84]. The thermal and mechanical characterization of the nanocellulose–MONPs nanohybrids has shown that the simultaneous achievement of an increased flexibility and toughness is still a challenging task for the design of more specialized and performing wound dressing materials.

## 6. Conclusions and Future Perspectives

Nanocellulose is a versatile material, providing high mechanical properties, low density, high crystallinity, biodegradability and biocompatibility at an affordable price. As a reinforcing agent in polymers or as a support to enhance the effectiveness of organic or inorganic NPs, nanocellulose gives rise to new materials which cover a huge range of properties and applications. Metal oxides, with their remarkable optical, electronic and magnetic characteristics were extensively studied to provide new functionalities to polymers. The combination of nanocellulose and metal oxide nanoparticles in new nanohybrids with biomedical applications is a promising platform for sustainable progress. In this review, the preparation methods, properties and applications of nanohybrids from nanocellulose and different metal oxides nanoparticles were presented. ZnO, TiO_2_, CuO, MgO or Fe_3_O_4_ NPs were studied to induce new antibacterial and magnetic functions to bacterial cellulose, cellulose nanofibrils or nanocrystals. In these hybrids, nanocellulose serves as a support material, providing flexibility and a high surface area for MONPs impregnation.

One of the challenging tasks in the synthesis of nanocellulose-MONPs nanohybrids is to obtain a nanolevel dispersion and high homogeneity. In the case of a high amount of MONPs, such as for magnetic nanomaterials, the homogenous dispersion is even more difficult to be obtained. Functionalization of nanocellulose by carboxylation [40], use of dispersion agents [95] or intensive mixing by ultrasound irradiation [53,55] are some of the methods attempted to improve the dispersion. Tailoring interfacial interactions and compatibility in these new organic–inorganic nanohybrids is of paramount importance. Therefore, new treatments are expected to be studied for improving dispersion and avoiding self-aggregation or microphase separation. Additionally, more eco-friendly processes and more efficient synthesis methods, adapted for the nanocellulose medium, are expected for these nanohybrids. The antibacterial or magnetic functionalities are enhanced with the increase of MONPs concentration in nanohybrids, however, toxicity also increases in the same direction and an optimum should be established in each case. In this context, the compatibility and interactions between NC and MONP should be better understood and used in the design of the new materials. Moreover, it is important to understand the influence of MONPs size, concentration, morphology and surface chemistry on the properties of nanohybrids and their toxicity. Specialized in vivo tests and detailed studies on animals are also necessary to establish the toxicity profile and the efficiency of these promising nanohybrids.

## Figures and Tables

**Table 1:** Various types of metal oxides incorporated in nanocellulose to obtain functional hybrids for biomedical applications.

Nr.	Nano-Cellulose	Metal Oxide NPs	Nanocomposite Preparation Method	Application	Ref.
1.	BC	ZnO	Ex situ synthesis of NPs, immersion of BC membrane and mixing	Wound dressing systems in burns complication	[47]
2.	BC	ZnO	MAPLE	Wound dressing materials	[51]
3.	BC	ZnO	Ex situ synthesis of NPs and mixing with BC dissolved in NMMO	Biomedical applications and bioelectroanalysis	[53]
4.	BC	ZnO	Ultrasonic-assisted in situ synthesis of NPs inside the BC template	Active antibacterial wound dressing	[55]
5.	BC	ZnO	Single-pot method: BC impregnation in NPs precursor	Wound healing	[59]
6.	BC	ZnO	SPP synthesis and deposition of NPs into BC pellicles	Antibacterial material in wound dressing	[63]
7.	BC	ZnO	BC modified with maleic anhydride template for in situ synthesis of NPs	Antibacterial wound dressing and tissue regeneration	[40]
8.	BC	TiO_2_	Ex situ sol-gel method	Antibacterial and photocatalytic applications	[77]
9.	BC	TiO_2_	Ex situ synthesis of NPs and mixing with BC dissolved in NMMO	Wound healing and tissue regeneration	[78]
10.	BC	CuO	GO-CuO nanohybrids blended with homogenized BC	Biomedical applications	[13]
11.	BC	MgO	Nanohybrids obtained by in situ co-precipitation method and ex situ incorporation of MgO-NPs in the BC	Clinical wound healing	[84]
12.	CNC	ZnO	Sheet-like CNC-ZnO nanohybrids by one-step hydrothermal method	Wound dressing	[88]
13.	CNF	Cu/CuO	In situ generation of Cu/CuO NPs using green reductive technique and coating CNF	Surgical bandage material	[89]
14.	BC	Fe_3_O_4_	In situ generation of Fe_3_O_4_ NPs inside the BC network in the presence of oleic acid or PEG	Tissue reconstruction at the cerebral aneurysmal neck defect	[95,97]
15.	CNC	Fe_3_O_4_	Ex situ generation of Fe_3_O_4_ and mixing with CNC-poly(citric acid) by ultrasonication	Dual contrast agent for MRI in biomedical applications	[104]
16.	CNC	CoFe_2_O_4_	In situ synthesis of CoFe_2_O_4_ NPs starting from precursor salts in the presence of CNC	Magnetic fluid hyperthermia, magnetically assisted drug delivery	[105]
